# Preparation, Optimization, and In Vivo Evaluation of Nanoparticle-Based Formulation for Pulmonary Delivery of Anticancer Drug

**DOI:** 10.3390/medicina55060294

**Published:** 2019-06-20

**Authors:** Nazimuddin Chishti, Satveer Jagwani, Dinesh Dhamecha, Sunil Jalalpure, Mohamed Hassan Dehghan

**Affiliations:** 1 Wockhardt Research Centre, D-4, M.I.D.C., Chikalthana, Aurangabad (M.S) 431006, India; nazimchishti@yahoo.com; 2 Dr. Prabhakar Kore Basic Science Research Centre, KLE Academy of Higher Education and Research, Belagavi 590010, Karnataka, India; satveer.mpharm@gmail.com (S.J.); dineshdhamecha@gmail.com (D.D.); jalalpuresunil@rediffmail.com (S.J.); 3 KLE University’s College of Pharmacy, KLE Academy of Higher Education and Research, Nehru Nagar, Belagavi 590010, Karnataka, India; 4 Department of Pharmaceutics, Y.B. Chavan College of Pharmacy, Zakaria Campus, Aurangabad (M.S) 431001, India

**Keywords:** PLGA nanoparticles, Lyophilization, non-small cell lung cancer, pulmonary

## Abstract

*Background and Oobjectives:* Lung cancer, a pressing issue in present-day society due to its high prevalence and mortality rate, can be managed effectively by long-term delivery of anticancer agents encapsulated in nanoparticles in the form of inhalable dry powder. This approach is expected to be of strategic importance in the management of lung cancer and is a developing area in current research. In the present investigation, we report on the formulation and characterization of docetaxel inhalable nanoparticles as a viable alternative for effective treatment of non-small cell lung cancer as a long-term delivery choice. *Materials and Methods:* Poloxamer (PLX-188) coated poly(lactic-co-glycolic acid) (PLGA) nanoparticles containing docetaxel (DTX-NPs) were prepared by simple oil in water (o/w) single emulsification-solvent evaporation process. The nanoparticles were collected as pellet by centrifugation, dispersed in mannitol solution, and lyophilized to get dry powder. *Results:* Optimized DTX-NPs were smooth and spherical in morphology, had particle size around 200 nm, zeta potential around −36 mV, and entrapment efficiency of around 60%. The in vitro anticancer assay was assessed and it was observed that nanoparticle-based formulation exhibited enhanced cytotoxicity when compared to the free form of the drug post 48 h. On examining for in vitro drug release, slow but continuous release was seen until 96 h following Higuchi release kinetics. DTX-NPs were able to maintain their desired characteristics when studied at accelerated conditions of stability. *Conclusions:* In-vivo study indicated that the optimized nanoparticles were well retained in lungs and that the drug level could be maintained for a longer duration if given in the form of DTX-NPs by the pulmonary route. Thus, the non-invasive nature and target specificity of DTX-NPs paves the way for its future use as a pulmonary delivery for treating non-small cell lung cancer (NSCLC).

## 1. Introduction

Administration of drug loaded nanoparticles by inhalation route is being exploited far and wide for delivery of chemotherapeutics [1,2,3], insulin, proteins and peptides, antibiotics, vaccines, etc. [4,5]. Inhalable nanoparticles have been a useful approach for systemic drug delivery such as in pain management, brain targeted drug delivery, as well as treatment of lung specific diseases [6,7,8]. Inhalable nanoparticles is not only a potential substitute for other delivery systems/routes of administration but also a favorable one owing to its localized affect, enhanced bio-availability, rapid onset, and possibility of self-administration apart from being non-invasive [1,2,9].

Lung cancer, a burning issue in present-day society with its high prevalence and mortality rate, can be managed effectively by long-term delivery of anticancer agents encapsulated in nanoparticles in the form of inhalable dry powder. This approach is expected to be of strategic importance in management of lung cancer and is a developing area in current research [10,11,12].

Localized delivery of anticancer drugs augment drug exposure of cancerous tumor localized in lungs as compared to intravenous administration and as a consequence improves pharmacological effects of these drugs [13]. Improved performance [14,15], optimized pharmacokinetics and pharmacodynamics [16], site-specific disease targeting of nanotechnology-based anticancer drugs can be realized by positively modulating the critical design parameters of nanoparticles and its nanoperiodic properties; i.e., relation between nanoparticle behavior and its fate in vivo [2,17,18,19].

Nevertheless, any pulmonary drug delivery system has to circumvent the body’s natural defense mechanism of mucociliary clearance and phagocytosis by alveolar macrophages. In addition, the delivery system should be designed such as to allow maximum deposition of formulation in middle and deep lung regions and this is where nanoparticles have forte [20]. Inhalable nanoparticles selectively get deposited in tumor tissue with marginal deposition in healthy tissue on account of impaired clearance of macromolecules and lipids from tumor, which enhances dwelling time of nanoparticles containing drug in tumor interstitial tissue [21,22,23].

Deposition of nanoparticle formulation in lungs, although critical, does not alone promise effective and long term delivery of inhaled drugs and needs regulation of the fate of deposited particles possibly by surface modification to evade the body’s natural immune response [2]. Polymeric nanoparticles find inherent advantage in confronting bio-barriers due to their size. Coating of these polymeric nanoparticles with hydrophilic stabilizer such as Poloxamer can aid nanoparticles to evade the bio-barriers by allowing swift diffuse through mucus and escape pulmonary and immune clearance [2,24,25,26,27].

In the presented work, poly-lactic-co-glycolic acid (PLGA) nanoparticles of docetaxel were formulated for the treatment of non-small cell lung cancer. These polymeric nanoparticles were prepared with Poloxamer (PLX-188) as helper molecule to overcome physiological barriers and were lyophilized with cryoprotectant to form inhalable dry powder.

## 2. Materials and Methods

Docetaxel was obtained from Glenmark Pharmaceuticals (Mumbai, India). PLGA (lactide:glycolide ratio of 75:25—Resomer 752H) was a generous gift from Evonik Industries (Mumbai, India). Poloxamer 188 (PLX-188) was purchased from BASF (Mumbai, India) and mannitol was purchased from Sigma Aldrich (Mumbai, India). Organic solvents (ethyl-acetate, acetone, acetonitrile) were purchased from Fisher Scientific (Mumbai, India) and were of analytical grade.

### 2.1. Preparation of Docetaxel Nanoparticles (NPs)

DTX-NPs were prepared by simple oil in water (o/w) single emulsification-solvent evaporation process as per available literature with minor modifications to suit laboratory conditions [28]. In brief, PLGA and docetaxel were solubilized in 2 mL of organic phase. Internal phase thus formed was added slowly to 2 mL aqueous surfactant solution (external phase) under continuous vortexing and after complete addition of oil phase to this aqueous phase, vortexing was continued for 15 s and then probe sonicated/homogenized without delay. Then, formed nanoparticle suspension was poured into external phase under stirring for hardening of nanoparticles. Consequently, organic solvent was allowed to evaporate overnight. The nanoparticles were collected as pellet by centrifugation at 17,000 rcf for 15 min (Kubota 6500, Tokyo, Japan) at 4 °C. The supernatant was discarded with care so as not to disturb the nanoparticle pellet and washed thrice to remove un-entrapped docetaxel and residual surfactant. The pellet was then dispersed in 5% mannitol solution using bath sonicator and frozen at −80 °C before it was lyophilized for 48 h using the optimized lyophilization cycle (Christ, Osterode, Germany) [29].

### 2.2. Risk Identification: Fishbone (Ishikawa) Diagram

A risk assessment of the material attributes and critical process parameters was performed to assess the impact that each attribute/parameter could have on the drug product CQAs (Critical quality attributes). Based on available scientific knowledge in literature, average particle size, type of dispersion (polydispersity index), zeta potential, and entrapment efficiency were identified as the CQAs of DTX-NPs due to their potential to impact the therapeutic properties and physical stability of the drug delivery system based on nanoparticles. The fishbone diagram helped in identification of eight variables of process and formulation with a potential to impact the properties of NPs and these variables were studied during development and optimization studies.

### 2.3. Screening of Formulation and Process Variables Using Plackett–Burman Design

For screening formulation and process parameters which may have an impact on the CQAs of DTX-NPs, trials based on Plackett–Burman statistical design were undertaken. Twelve runs were executed to study eight identified factors. In each trial, the level of parameter was selected based on pilot trials as well as literature findings. Organic/aqueous phase ratio of 1:2 during the emulsification step was fixed based on literature [28]. The selected factors with levels are provided in Table 1. Randomized design of experiments was generated and analyzed statistically utilizing Design expert^®^7 software (State-Ease, Minneapolis, MN, USA) as shown in Table 2.

The statistical methods like multi-linear regression analysis and analysis of variance were used to test significance of model. Experimental trials were taken in triplicate. Average or mean particle size (Y_1_), zeta potential (Y_2_), entrapment efficiency (Y_3_), and polydispersity index (Y_4_) were the CQAs or dependent variables.

### 2.4. Optimization Studies—Box–Behnken Design

Post screening and identification of critical process and formulation variables, and preparation of DTX-NPs, was optimized by taking trials generated by the Box–Behnken design of experiment. Three factors were studied at 3 levels. Results obtained in screening trials were used to set the levels in the Box–Behnken design of experiment as shown in Table 3.

Levels of five factors evaluated in screening trials were fixed in optimization studies as shown in Table 4, as the effect of some of these factors on dependent variables was observed to be statistically insignificant or results achieved utilizing one of the two levels of these factors were much favorable as compared to the other level.

Randomized design of experiments was generated as shown in Table 5 and analyzed statistically utilizing Design expert^®^7 software. The trials were taken in triplicate.

For each of the measured response, regression analysis was performed and the established polynomial equation is provided below:

(1) $$Y=\alpha_{0}+\alpha_{1}X_{1}+\alpha_{2}X_{2}+\alpha_{3}X_{3}+\alpha_{12}X_{1}X_{2}+\alpha_{13}X_{1}X_{3}+\alpha_{23}X_{2}X_{3}+\alpha_{11}X_{1}^{2}+\alpha_{22}X_{2}^{2}+\alpha_{33}X_{3}^{2}$$

where Y denotes response, X_1_ to X_3_ denotes main effects of factors, X_1_X_2_, X_1_X_3_, X_2_X_3_ denote interaction effects of factors, $X_{1}^{2}$, $X_{2}^{2}$, $X_{3}^{2}$ denote quadratic effects of factors, α_0_ denotes constant, and α_1_–α_3_ denotes coefficients of the factors. The *p* values of the regression coefficients (and α_1_–α_3_) were obtained to evaluate the significance of independent variables on dependent variables. Significance of the model was assessed by ANOVA.

The process and formulation parameters were optimized in order to obtain desired average particle size (Y_1_), zeta potential (Y_2_), entrapment efficiency (Y_3_), and polydispersity index (Y_4_) of DTX-NPs by determining the levels of concentration of surfactant (X_1_), amount of PLGA in organic phase (X_2_), and number of sonication cycles (X_3_). To achieve this, a design space was built and the optimized DTX-NPs were fabricated in order to evaluate the relation between actual and predicted values. Physicochemical properties of the optimized formulation were then characterized.

### 2.5. Characterization

#### 2.5.1. Particles Size, Polydispersity Index (PDI) and Zeta-Potential

Mean particle size, zeta potential and polydispersity index (PDI) of DTX-NPs were analyzed using Malvern Zeta Sizer Instrument (Malvern, Worcestershire, UK). The pellet obtained after centrifugation was re-suspended in deionized water before the analysis.

#### 2.5.2. Entrapment Efficiency

For estimation of the percentage of drug entrapped in DTX-NPs, reported HPLC method was used with minor modifications. In brief, supernatant obtained after centrifugation of formulation suspension was discarded. The pellet obtained as sediment was taken and solubilized in acetonitrile. Analysis was carried out by HPLC (Shimadzu, Kyoto, Japan) consisting of SPD-M20A diode array detector and Luna C18 column (Phenomenex Inc., Torrance, CA, USA).

Acetonitrile and orthophosphoric acid (OPA) (58:42) were the mobile phase pumped at a flow rate of 1.0 mL/min with constant column temperature of 37 °C. Ten µL was the injection volume with docetaxel eluting at 7.4 min with λ-max 230 nm.

#### 2.5.3. Morphology of Nanoparticles

To scrutinize surface texture of DTX-NPs, scanning electron microscopy (SEM) Field Electron and Ion Company (FEI), Quanta 200 (FEI, Hillsboro, OR, USA), Accelerating Voltage 1.0 kV, high vacuum was used. Pellet of DTX-NPs was re-dispersed in deionized water and were charged for analysis. Transmission electron microscopy (TEM) Philips, CM200 (Philips, Hillsboro, OR, USA), 20–200 kV, resolution 2.4 Angstrom was used for studying the texture. Re-dispersed nanoparticles were dropped into carbon-coated copper grids and were allowed to dry completely. Sample staining was carried out by 2% w/v uranyl-acetate and images were captured.

#### 2.5.4. Fourier-Transform Infrared Spectroscopy (FTIR)

The FTIR transmission spectrum of samples (PLGA, docetaxel, and lyophilized DTX-NPs) was obtained using a Shimadzu spectrophotometer (Shimadzu, Kyoto, Japan). KBr pellet method was used and each KBr disk was then scanned. The characteristic peaks for different samples were recorded.

#### 2.5.5. Differential Scanning Calorimetry (DSC) Analysis

Lyophilized DTX-NPs, pure docetaxel, PLGA and PLX-188 were analyzed on Differential scanning calorimeter, Shimadzu (Shimadzu, Kyoto, Japan). Heating rate was 10 °C/min and scanning temperature ranging from 0 to 200 °C.

#### 2.5.6. X-ray Diffraction (XRD) Analysis

X-ray diffractometer, PANalytical and X’Pert Pro MPD (Malvern, Worcestershire, UK) was used to generate the XRD pattern for pure docetaxel and lyophilized formulation. Analysis was performed at a voltage of 45 kV and 40 mA. The scanned angle (2θ) was set from 3–40°, and scanning rate was 0.02° per 25 s.

#### 2.5.7. In-Vitro Release of Docetaxel

Optimized DTX-NPs batch 473 mg (equivalent to 4 mg of entrapped drug) was studied for release pattern in vitro. Phosphate buffer saline (PBS) with 0.5% polysorbate-80 as surfactant and pH 7.4 was used as release media maintaining sink. Ten mL release media was taken in tubes made up of polypropylene and DTX-NPS were suspended in it. Tube assembly was immersed in water bath maintained at a temperature of 37 °C under stirring with magnetic stirrer at 350 rpm. Aliquots were taken at defined intervals, filtered and analyzed by HPLC.

#### 2.5.8. In Vitro Anticancer Assay

A549 lung cancer cell lines were introduced to Dulbecco’s modified Eagle’s medium (DMEM, HiMedia, Nashik, India) contained in micro titer plate with 96 well at 1 × 10^4^ cells/well density. The assembly was incubated for 24 h at 37 °C and 95% humidity with CO_2_ environment in order to allow attachment. Cell count determination was done by haemocytometer. DMEM was then removed and fresh medium (0.2 mL) was added containing different concentrations of free docetaxel and DTX-NPs dose equivalent to 40, 20, 10, 5, 2.5 and 1.25 nM followed with incubation at 37 °C for 48 h. Subsequently, medium from each well was withdrawn and 20 µL of methyl-thiazolyl-tetrazolium (MTT) solution was replenished to wells. Assembly was allowed to incubate at 37 °C for 4 h. This was followed by addition of 100 µL DMSO to each plate well and vortex for 15 min in order to dissolve formazan crystals. The absorbance was noted using ELISA reader at 570 nm filter and the percentage of viable cells was calculated. Human lung cancer cell line (A549) experiment results were plotted using graph pad prism version 7.0 for estimation of IC_50_ value of DTX-NPs. The IC_50_ value of DTX-NPs was expressed as mean ± SD.

#### 2.5.9. Stability Studies

DTX-NPs were charged on accelerated conditions to assess stability. For this, lyophilized formulation was filled in vials and sealed with rubber plugs and flip off seal and was kept stable for 3 months at temperature of 25 ± 2 °C and RH 60 ± 5%. The stability tests performed were physical description, particle size, PDI and drug content.

#### 2.5.10. In Vivo Studies

In vivo studies were carried out on rats (weighing ~ 200 g) after necessary approval from Institutional Animals Ethical Committee for the protocol (CPCSEA/IAEC/P’ceutics-31/2016-17/128, date of approval: August 2017). At each time point, 3 animals were studied. Rats were grouped into Group 1, in which free drug powder (DTX) was administered, and Group 2, in which lyophilized DTX-NPs were administered after passing through sieve no. 200 (ASTM) by the tracheal pulmonary method (tracheotomy). After anesthesia, the animals were placed in supine position and the neck was extended to facilitate tracheal incision. The incision was made in the midline below the neck. After viewing the trachea, an incision was made to insert the cannula between the fourth and the fifth tracheal ring.

Tissue homogenate study was performed to determine organ distribution and lung pharmacokinetics. Rats were sacrificed at 30 min, 2 h, 8 h, 12 h, 18 h and 24 h. Homogenates of different tissues like lungs, liver, kidney, and spleen (20% w/v) were analyzed for level of drug. Acetonitrile (ACN) was used to deproteinize the homogenated tissues and centrifuged so as to obtain supernatant which was analyzed for DTX content. Lung concentration v/s time profile was obtained and C_max_, T_max_ were estimated. Mean residence time (MRT), elimination half-life t^1/2^ and concentrations of drug in lungs over the duration (AUC_0-∞_) were estimated using Phoenix WinNonlin software (Certara, Princeton, NJ, USA).

#### 2.5.11. Lyophilization Optimization Studies

The initial step for optimizing the freeze-drying cycle was the identification of the critical process parameter required for freeze-drying, i.e., freezing temperature and the collapse temperature. This was done with the help of BTL Lyostat-2 freeze-drying microscope (FDM) (Biopharma Technology Ltd., Winchester, UK). The determination of lyophilization characteristics for the solution was carried out using the Biopharma Lyostat-2 FDM, equipped with Linksys image and data capture software. A 2 μL sample of the product was placed between cover slips and frozen to −40 °C. The freezing temperature and the collapse temperature were thus determined.

## 3. Results

### 3.1. Risk Assessment: Ishikawa Diagram

**Figure 1:**
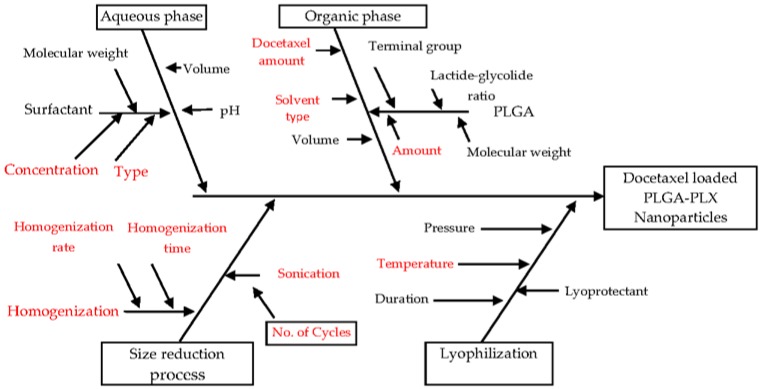
Ishikawa diagram demonstrating process and formulation variables which may impact the Critical Quality Attributes (CQAs) of nanoparticles.

The fishbone diagram was used to determine potential risks of the process and formulation variables on the CQAs of DTX-NPS, viz. mean particle size, PDI, zeta potential and encapsulation efficiency as shown in Figure 1. Based on prior knowledge and screening experiments, eight potential risk factors were identified and were assessed in experimental designs.

#### Risk Assessment: Plackett–Burman Design of Experiment

With an objective of screening the most significant process and formulation variables, the Plackett–Burman statistical experiment tool was utilized. Twelve experiments were conducted to study each of the eight factors at two levels. The details are provided in Table 6.

Amount of PLGA in organic phase (X_2_), surfactant concentration (X_3_), surfactant type (X_4_), size reduction process (X_5_), and solvent type (X_6_) are the significant factors effecting average particle size (Y_1_) (Table 7). *R*^2^ value was 0.9940 and *p* value was 0.0269 indicating a significant fit.

For zeta-potential (Y_2_), surfactant concentration (X_3_), surfactant type (X_4_) and the size reduction process (X_5_) were the most significant factors (Table 7). R^2^ value was 0.9999 and *p*-value was 0.0279, and hence, a significant fit statistically.

For the entrapment efficiency (Y_3_), the most significant factors were the docetaxel amount in the organic phase (X_1_), amount of PLGA in the organic phase (X_2_), concentration of surfactant (X_3_) and surfactant type (X_4_). The size reduction process (X_5_) was considered almost insignificant with *p*-value 0.0491 (Table 7). R^2^ value was 0.9955 and *p*-value was 0.0200 indicating a significant fit. Critical parameters were further studied in the Box–Behnken design.

### 3.2. Optimization Studies: Box–Behnken Design of Experiment

Subsequent to the determination of the critical parameters for formulation and process in screening trials, three factors were studied at three levels utilizing Box–Behnken design to understand the effects of surfactant concentration in aqueous phase (X_1_), the amount of PLGA in the organic phase (X_2_) and sonication time (X_3_) on average particle size, zeta potential, entrapment efficiency and polydispersity index of DTX-NPs. Results are given in Table 8. 

The correlation coefficients of factors and corresponding *p*-values are provided in Table 9. Factors for which *p*-values were less than 0.05 were termed significant.

The determination coefficient (R^2^) for the observed and the predicted values were assessed for testing significance of the model. R^2^ for mean particle size, zeta potential, encapsulation efficiency and polydispersity index were 0.9616, 0.8662, 0.9300, and 0.8712, respectively.

The *p*-values obtained after ANOVA were 0.0004, 0.0223, 0.0028, and 0.0198 for average particle size, zeta potential, encapsulation efficiency, and polydispersity index, respectively indicating that the relating responses can be predicted with precision using the mathematical model thus established.

The factors with significant impact on mean particle size of DTX-NPs were the concentration of surfactant and amount of PLGA added to organic solvent.

As the amount of PLGA increases there was an increase in average particle size value while increasing concentration of surfactant increased the average size of nanoparticle. Interaction of surfactant concentration-PLGA amount and surfactant concentration-sonication cycle were significant. Quadratic effect for the PLGA amount was significant. Similar outcomes were reported in literature [30,31,32,33,34,35,36].

Mean particle size can be estimated by following the established polynomial equation with order of second degree:

(2) $$Y=241+25.59X_{1}+58.67X_{2}-2.86X_{3}-57.35X_{1}X_{2}-23.88X_{1}X_{3}-9.80X_{2}X_{3}+4.36X_{1}^{2}+22.49X_{2}^{2}+7.51X_{3}^{2}$$

For zeta potential, the most significantly impacting factor was concentration of surfactant.

Increasing surfactant concentration shifts the zeta potential towards the neutral side. Interaction of surfactant concentration-PLGA amount and quadratic effect for PLGA amount were significant. Similar outcomes were reported in literature [30,31,32,33,34,35,36].

Zeta potential can be estimated by following the established polynomial equation with order of second degree:

(3) $$Y=-21.56+7.88X_{1}-2.67X_{2}+2.21X_{3}-16.73X_{1}X_{2}-1.45X_{1}X_{3}-3.6X_{2}X_{3}+0.49X_{1}^{2}+18.55X_{2}^{2}-7.83X_{3}^{2}$$

The concentration of surfactant (X_1_), amount of PLGA (X_2_), and number of sonication cycles (X_3_) were the most significantly impacting factors for entrapment efficiency. Quadratic effect of surfactant concentration (X_1_) was also significant.

Increasing the PLGA amount and number of sonication cycles had a beneficial impact on efficiency of the encapsulating drug, while increasing the concentration of surfactant diminished entrapment efficiency. Quadratic effect of surfactant concentration was also significant. Similar outcomes were reported in earlier studies [30,31,32,33].

Entrapment efficiency can be estimated by following the established polynomial equation with order of second degree:

(4) $$Y=54.14-8.08X_{1}+10.78X_{2}+5.78X_{3}+0.72X_{1}X_{2}-4.31X_{1}X_{3}+3.49X_{2}X_{3}+5.52X_{1}^{2}-3.36X_{2}^{2}+3.92X_{3}^{2}$$

For polydispersity index (Y_4_), surfactant concentration (X_1_) was most significant factor. Increasing surfactant concentration gave better polydispersity index. In addition, interaction of surfactant concentration and PLGA amount along with quadratic effect of the PLGA amount were significant [30,31,32,33].

Polydispersity index can be estimated by following the established polynomial equation with order of second degree:

(5) $$Y=0.18+0.037X_{1}-0.012X_{2}-0.023X_{3}-0.089X_{1}X_{2}-0.021X_{1}X_{3}+5.25\times 10^{-3}X_{2}X_{3}-0.023X_{1}^{2}+0.055X_{2}^{2}+0.025X_{3}^{2}$$

**Figure 2:**
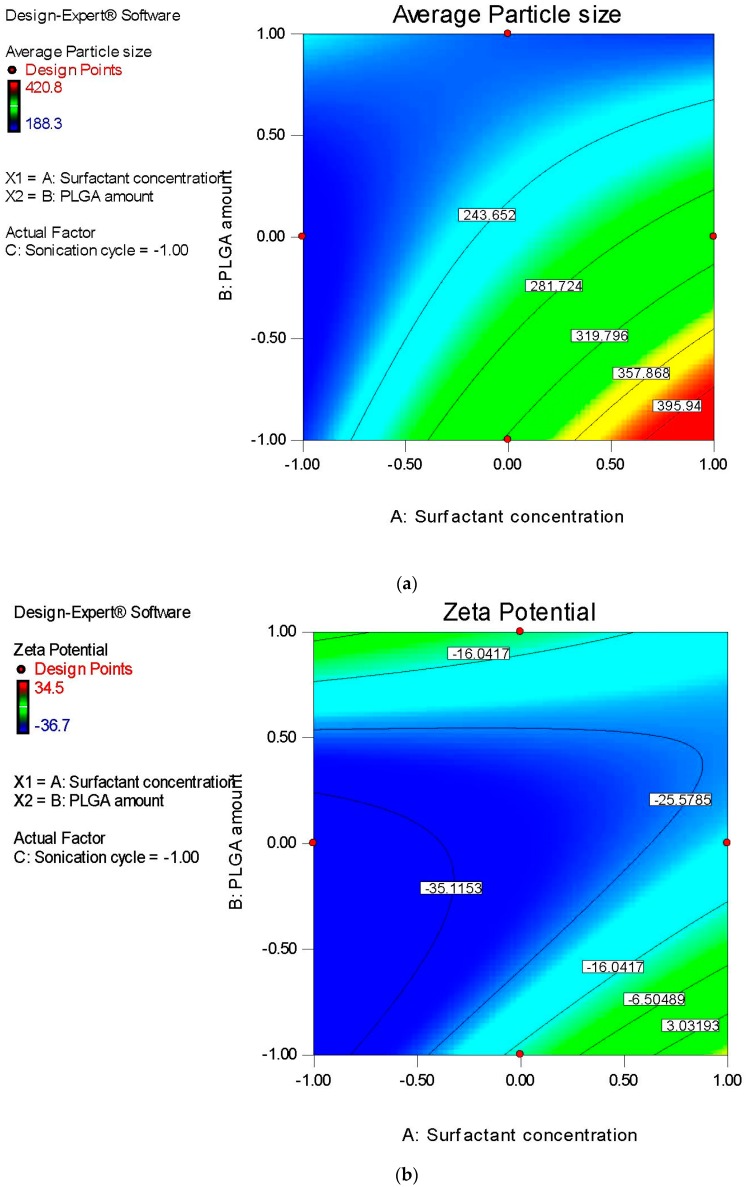

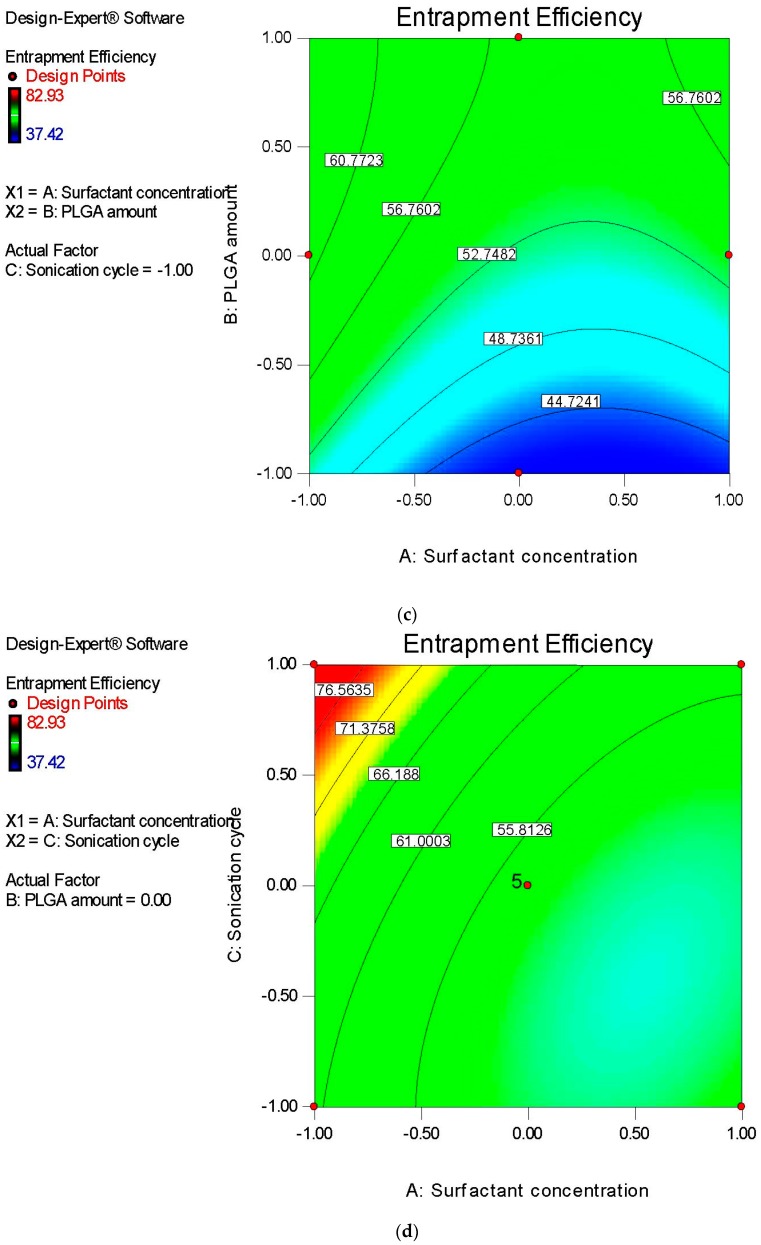

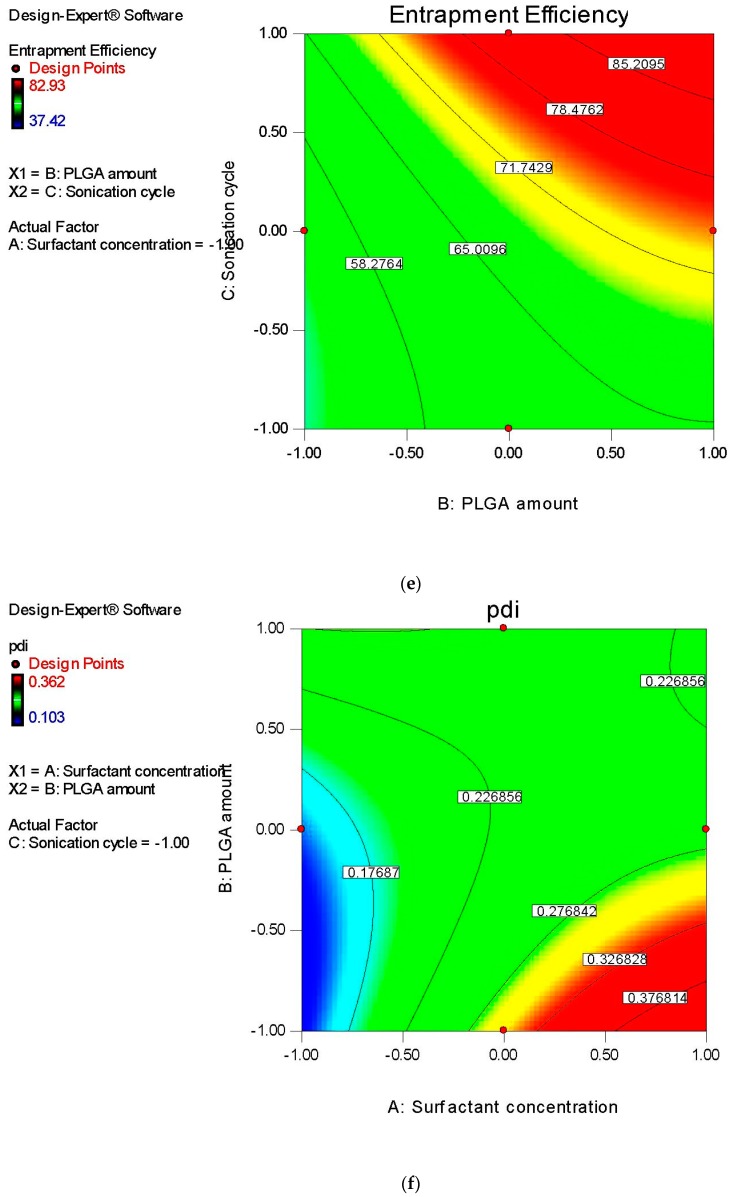
Contour plots presenting impact of: (**a**) Concentration of surfactant and amount of poly (lactic-co-glycolic acid) (PLGA) on mean nanoparticle size, (**b**) surfactant concentration and PLGA amount on zeta potential, (**c**) surfactant concentration and PLGA amount on the encapsulation efficiency, (**d**) surfactant concentration and sonication time on the encapsulation efficiency, (**e**) PLGA amount and sonication time on the encapsulation efficiency, and sonication time, and (**f**) surfactant concentration and PLGA amount on polydispersity index of docetaxel nanoparticles.

Factor effects were thoroughly evaluated by visual presentation of results obtained for mean particle size and encapsulating efficiency in contour plots (Figure 2).

**Figure 3:**
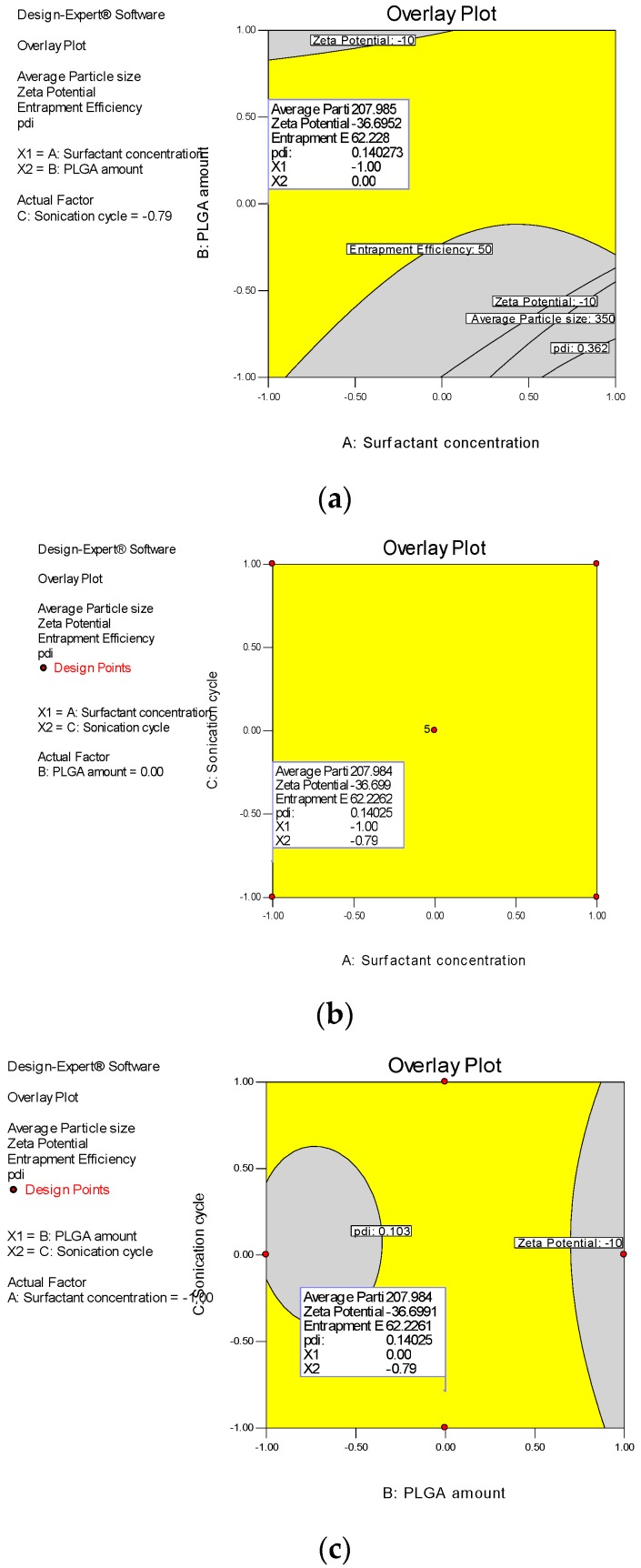
Design space for DTX-NPs. Yellow regions show the possible combination to attain expected results for average nanoparticle size (200–350 nm), zeta potential (<−10), encapsulation efficiency (>50%) and polydispersity index (0.103–0.362). (**a**) Overlay plot of surfactant concentration and PLGA amount, (**b**) overlay plot of surfactant concentration and sonication cycle and (**c**) overlay plot of sonication cycle and PLGA amount.

The design space for DTX-NPS was established with target of a mean nanoparticle size lower than 350 nm, upper limit of zeta potential as −10, entrapment efficiency higher than 50% and polydispersity index in the observed range. For this, overlaid contour plots including responses were created (Figure 3).

In order to find out the optimum formula of DTX-NPs, desirability function (d value) was built based upon target response. D value near to “1” signifies desirable set of results and “0” is detrimental. Average particle size (200–350 nm), zeta potential (−10 to −37), maximum entrapment efficiency (>50%), and polydispersity index in entire observed range were set as constraints.

Based on the design space, surfactant concentration and sonication time was fixed at the lowest point and amount of PLGA was maintained at medium point. Thus, with this set of pattern, a desirability value of 0.967 was achieved. For optimum formulation, Table 10 shows the observed and predicted value.

#### 3.2.1. Lyophilization Cycle Optimization

**Figure 4:**
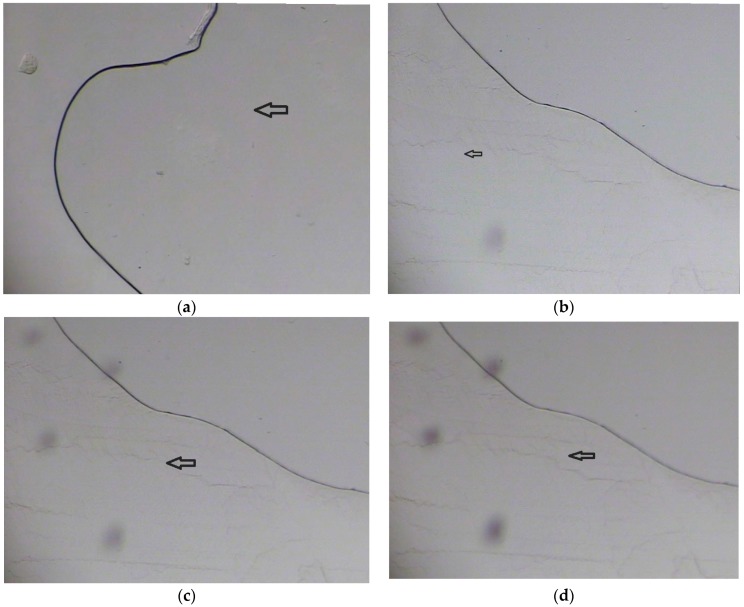

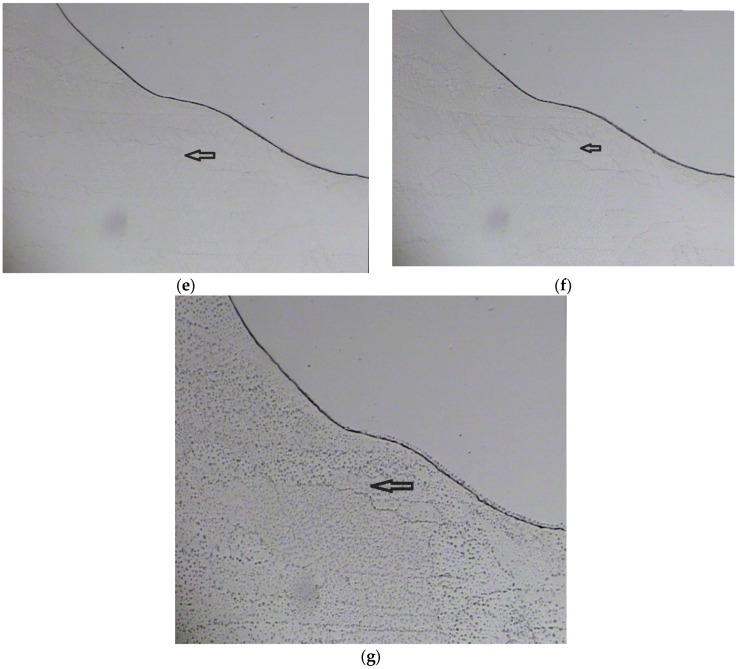
Sequential images of freeze-drying microscopy: (**a**) At temperature −11.6 °C, (**b**) at temperature −27.8 °C, (**c**) at temperature −33.4 °C, (**d**) at temperature −40.5 °C, (**e**) at temperature −15.0 °C, (**f**) at temperature −10.3 °C, (**g**) at temperature −0.6 °C.

Based on the images (Figure 4) taken with the help of freeze-drying microscope, the freezing and collapse temperatures were found to be −28 to −32 and −15 to −10 respectively.

Therefore, it was decided to keep the temperature well below the collapse temperature during primary drying to evade collapse of the cake structure. The optimized lyophilization cycle is shown in Table 11 below.

#### 3.2.2. FTIR Spectroscopy

**Figure 5:**
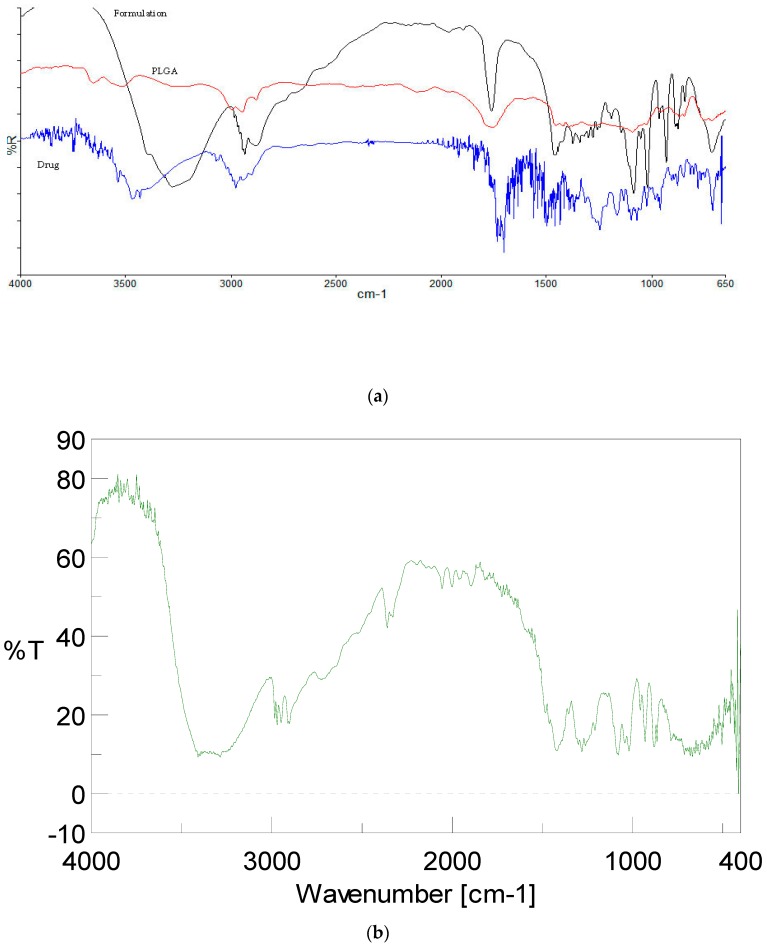
FTIR spectra of (**a**) formulation, polymer and docetaxel drug, (**b**) mannitol.

FTIR spectroscopy study was performed to investigate any interaction in between docetaxel drug, PLGA polymer, and DTX-NPs formulation (Figure 5).

Spectrum of docetaxel showed characteristic peaks attributable to O–H and N–H stretching vibrations at 3467 cm^−1^ (Strong and broad band), C–H stretching vibrations (medium and broad band stretch in CH_3_ and CH_2_ groups) at around 2900 cm^−1^, ester and keto C=O vibrations at 1722–1704 cm^−1^ (multiple strong bands), C–C stretch in aromatic groups at 1494 (medium band), C–O asymmetric stretch in ester groups at 1248 cm^−1^ (strong bands), C–O symmetric stretch in ester groups at 1098 cm^−1^ (medium band) and C–OH stretch in alcohol group at 1072–1098 cm^−1^ (multiple medium bands). FTIR spectrum of PLGA showed peaks at 3524.98 cm^−1^ (O–H stretch), 1759.46 cm^−1^ (ester group), 1396.21 cm^−1^ (bending C–H vibrations), and 1092.22 cm^−1^ (C–O stretch). N–H stretching vibrations at 3467 cm^−1^, ester and keto C=O stretching vibrations at 1722–1704 cm^−1^ were absent in the spectrum of DTX-NPs, which might be due to complete encapsulation of docetaxel into the PLGA-NPs. The peak which is observed in the 3300–3400 region appears to be of mannitol used as lyoprotectant.

#### 3.2.3. X-ray Diffractometry (XRD)

**Figure 6:**
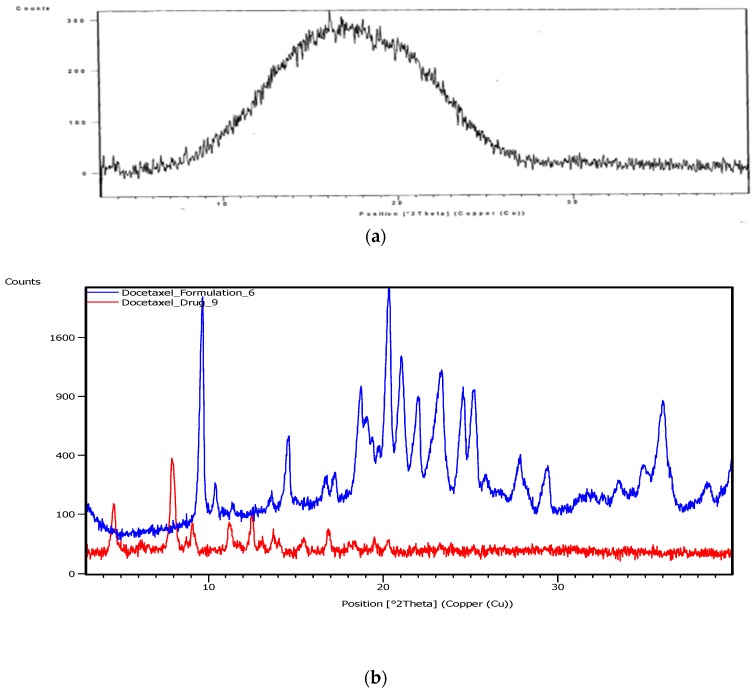
X-ray Diffraction (XRD) patterns of (**a**) PLGA, (**b**) docetaxel and DTX-NPs.

To find out the nature (amorphous or crystalline) of docetaxel entrapped into/onto the nanoparticles, XRD patterns of docetaxel and DTX-NPs were studied. The diffractogram of docetaxel showed a prominent peak at the 2θ value of 7.9°, however such distinct peak was absent in the diffractogram of DTX-NPs (Figure 6).

#### 3.2.4. Differential Scanning Calorimetry (DSC)

**Figure 7:**
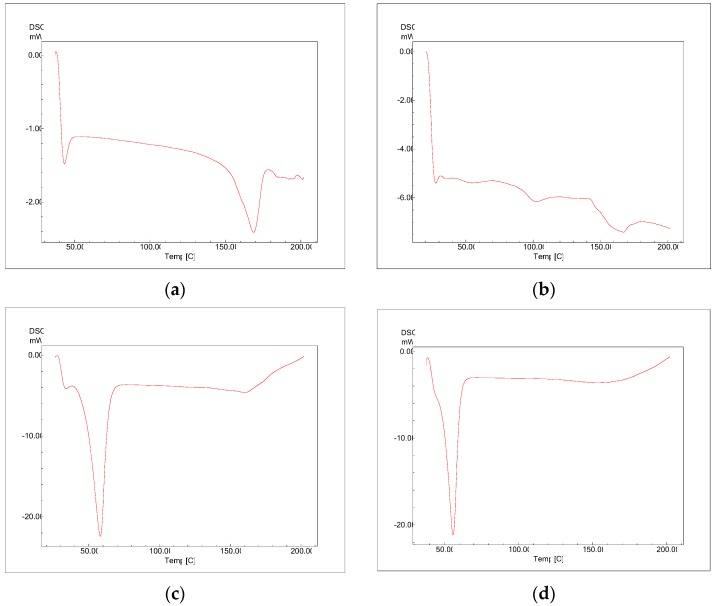
Differential Scanning Calorimetry (DSC) thermograms of (**a**) docetaxel, (**b**) PLGA, (**c**) PLX-188, (**d**) DTX-NPs.

The physical status of docetaxel in the PLGA-NPs was studied by DSC analysis. Figure 7 reflects DSC thermogram of pure docetaxel, PLGA, PLX, and lyophilized DTX-NPs (without mannitol). In the DSC thermogram of docetaxel, a sharp endothermic peak of melting at 166 °C was observed which indicates crystalline state. The PLGA thermogram exhibited glass transition temperature around 52 °C. The glass transition temperature for PLGA was not affected by the nanoparticles preparation procedure. PLX-188 thermogram exhibited endothermic peak at melting temperature of 57 °C. DTX-NPs did not show peaks related to the melting point of docetaxel which may be because of decreased docetaxel crystallinity in the formulations and/or drug solubilization in the polymeric matrix. Similar results were reported in literature by other authors when a hydrophobic drug was encapsulated in PLGA-NPs [31].

#### 3.2.5. Scanning Electron Microscopy and Transmission Electron Microscopy

**Figure 8:**
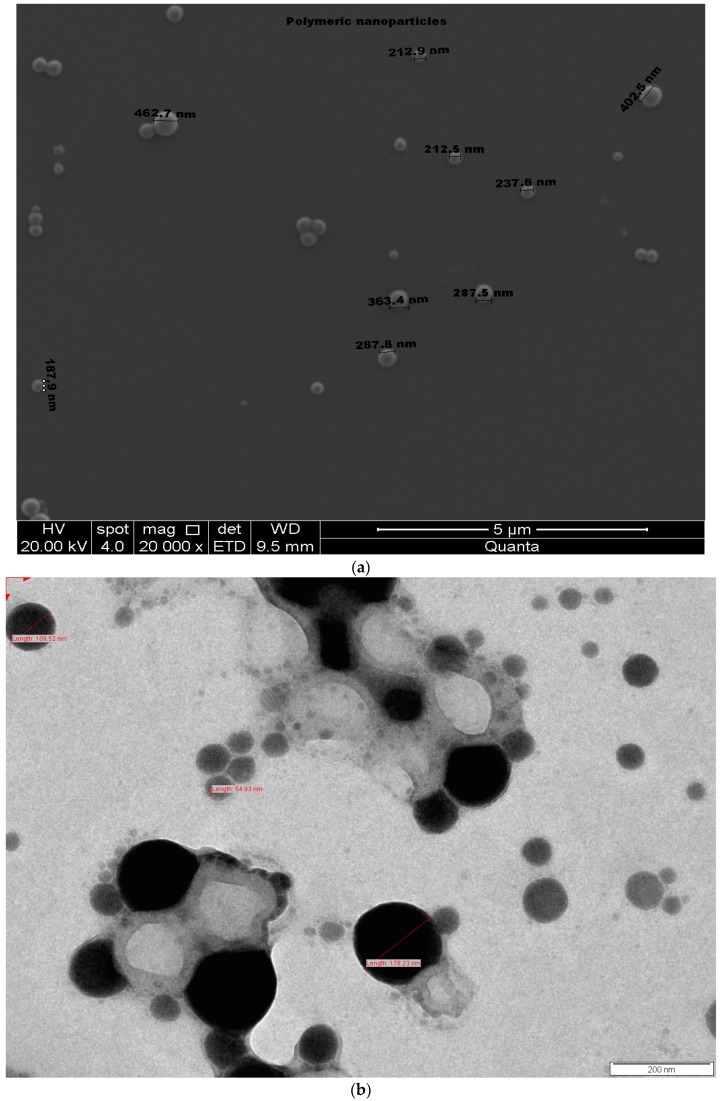

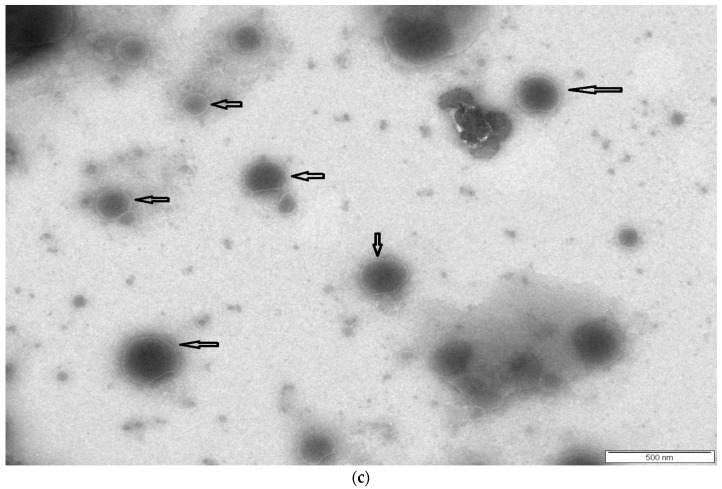
Surface morphology (**a**) SEM image, (**b**) TEM image, (**c**) Layer of PLX in TEM image.

The SEM and TEM images showed prepared DTX-NPs are homogenous, possess smooth and spherical surfaces without aggregation (Figure 8).

#### 3.2.6. In Vitro Drug Release

**Figure 9:**
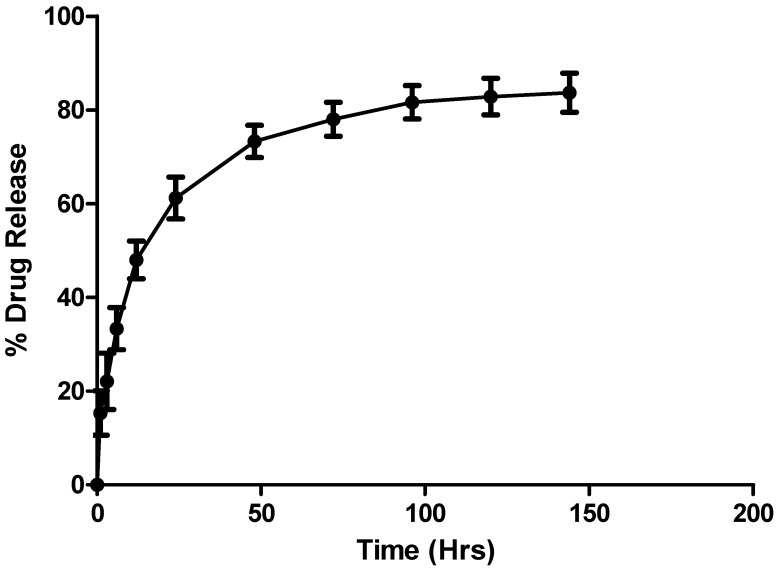
In vitro drug release profile of docetaxel from DTX-NPs.

Optimized DTX-NPs formulation was studied for in vitro drug release profile (Figure 9).

As reported in literature, drug release from PLGA-NPs illustrates biphasic patterns with primary burst release attributable to dissolution of docetaxel adsorbed on the surface of NPs. Subsequently, slow but continuous release was seen until 96 hours. The release kinetics of drug from NP formulation was studied as per zero and first order, Higuchi, Peppas and Hixson-Crowell models. The Higuchi model showed best fit with determination coefficient as R^2^ = 0.90. The release from PLGA polymer may be controlled by diffusion and matrix erosion [30,31,32,33,37].

#### 3.2.7. In Vitro Anticancer Assay

**Figure 10:**
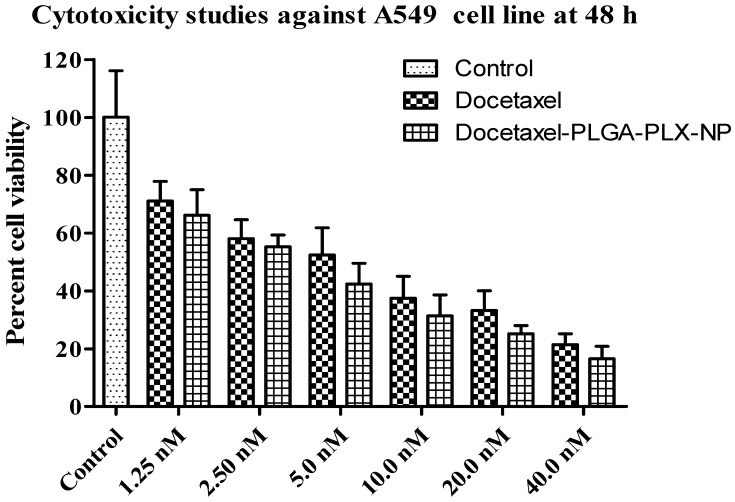
Cytotoxicity studies against A549 cell line. The data are means ± SD (n = 3).

In vitro cell cytotoxicity study was performed on A549 cells, cell viability after the treatment with plain docetaxel and DTX-NPs at different concentration, after 48 h. IC_50_ was determined statistically [38]. One way ANOVA followed by “Dunnett’s Multiple Comparison Test” has been used to access the statistical comparison (Figure 10).

#### 3.2.8. Stability Studies

Lyophilized nanoparticles containing 5% mannitol as a cryoprotectant were charged on accelerated stability conditions. At the end of the storage period, lyophilized DTX-NPs were analyzed and it was observed that there was no melt back of cake and the product was stable. There was no significant change in description, content of entrapped drug, particle size, and PDI as compared to initial time point (Table 12).

#### 3.2.9. In Vivo Studies

**Figure 11:**
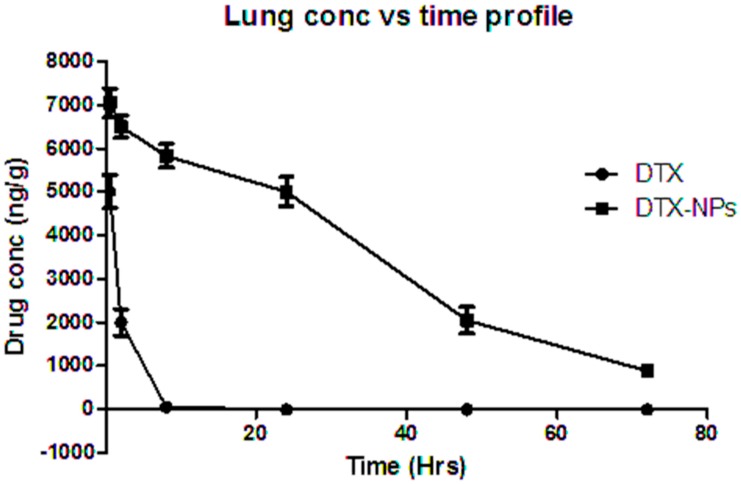
In vivo lung retention study performed in rats for free drug and DTX-NPs.

In vivo studies were carried out on Wistar rats with administration of NP formulations via pulmonary route, and concentration of drug was examined in different organs like lung, spleen, liver and kidney). The HPLC bioanalytical method was used for analysis of docetaxel in the NPs. A comparative study of various pharmacokinetic parameters was done for DTX-NPs versus concentration of free drug in lungs at the same dose, using rats (n = 3). The lung concentration after pulmonary administration of plain drug powder and DTX-NPs utilizing tracheotomy technique was estimated by the HPLC method. DTX-NPs (885 ± 56.35 ng/g) were detectable even after 72 h in the lungs, as compared to free drug which was not detectable after approximately 8 h (Figure 11). Similar outcome was reported in literature [39].

Thus, it provided a clear difference between the lung concentrations of DTX-NP and free drug. This study indicated that nanoparticles were well retained in lungs and that the drug level could be maintained for a longer duration if given in the form of DTX-NP by the pulmonary route.

Non-compartmental analysis was performed for concentration of drug in lung vs. time to obtain pharmacokinetic parameters with the help of WinNonlin software and presented in Table 13.

**Figure 12:**
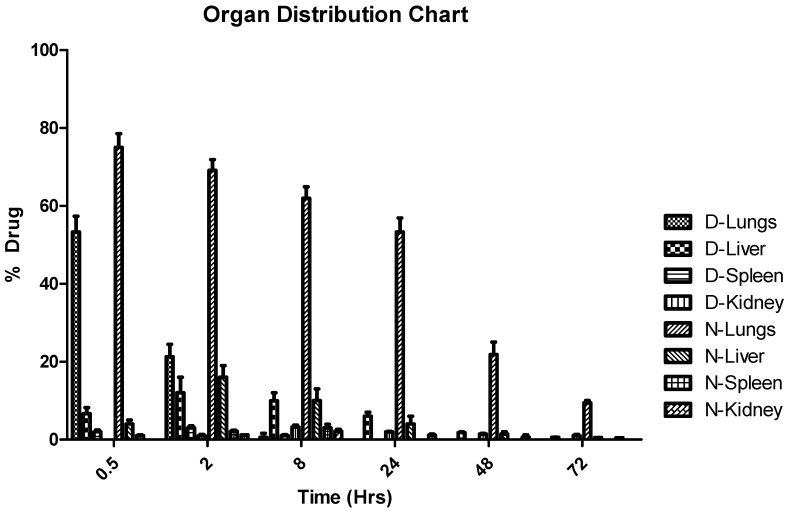
Organ distribution of free drug and DTX nanoparticles after intra-tracheal administration.

Free DTX and DTX-NP reached C_max_ at approximately 0.5 h. In contrast to pure DTX administration, DTX nanoparticles gave sustained release in lungs for more than 72 h, whereas pure DTX was cleared within 8 h. AUC increased significantly when DTX was given in the form of nanoparticles. The values of AUC_0-∞_ for free DTX was 10,069.58 ± 744.5 ng/g·min and 279,118.6 ± 6919.80 ng/g·min for DTX-NP. C_max_ was 5011.33 ± 379.85 ng/g for free DTX, whereas 7050.367 ± 334.93 ng/g for DTX-NP. Mean retention time for free DTX was 2.14 ± 0.31 whereas 34.30 ± 1.37 for DTX-NP which signified prolong drug residency in lungs (organ of interest). Hence, prepared formulation may be used to maintain the lung concentration for 72 h. The percent drug content in lungs, liver, spleen, and kidney of plain drug, and its nanoparticle form are shown in Figure 12 and Table 14.

## 4. Discussion

The aim of this study was to obtain an optimized nano-particulate composition that could be administered via pulmonary route. It was also expected that the formulation should be retained in the organ of interest (lungs) for a longer duration so as to have a higher concentration of drug. Considering the amount of powder that can be inhaled or a liquid suspension that can be administered exploring the pulmonary route, the encapsulation efficiency was anticipated to be high making it a CQA. In order for the nano-formulation to be effectively delivered to lungs via inhalation route, average particle size and polydispersity index were aimed to be optimized enabling the retention in lungs for an enhanced duration. Physical stability before the nanoparticle suspension gets lyophilized was also of importance. Hence, zeta potential was considered critical.

In general, while PLGA nanoparticles are formulated using emulsification solvent evaporation technique, and once the emulsification step is completed, the organic phase with PLGA and/or drug and aqueous-phase containing helper molecule/surfactant are in the state of thermodynamic equilibrium. Transferring the formed emulsion into water with surfactant destabilizes the equilibrium of this system. Due to this, the organic solvent diffuses to the water-phase and during this transport phenomenon, PLGA nanoparticles are formed, and the size of formed nanoparticles may rely upon the type of organic phase solvent used; the same was found to be a significant factor during screening performed in this study. Organic solvents which are partially soluble in water like EA and solvents which are fully soluble in water like AC were studied. It was observed that, in case of partially miscible solvent (EA), small particles around 200–400 nm in mean particle size were attained, while large particles around 400–900 nm in mean particle size were obtained using solvents which are fully soluble in water (AC). In addition, the suspension of PLGA nanoparticles formulated using EA was more transparent in appearance than that with AC. This shows that the organic solvent used plays a significant role in deciding the size of nanoparticles obtained. The interfacial tension in between the organic and aqueous phase which results from partial water solubility of EA and the capability of stabilizer to protect NPs against aggregation might be responsible for the small size of NP obtained with EA. On the other hand, complete miscibility of AC with water hinders the formation of stable emulsion and upon mixing the aqueous and organic phases, the polymer precipitates out as large particles of sub-micron size. Hence, EA was used for optimization studies [34].

In screening trials (PBD), NPs formulated with PLX-188 showed smaller size compared to formulations fabricated with PVA. PLX has higher HLB value (29) compared to PVA (18) and hence, PLX is able to stabilize even smaller particles in contrast to PVA [30,31,32,33,34,35,36].

Since both the surfactants were non-ionic, the nanoparticle formulations made exhibited negative zeta potential; magnitude was higher with PLX-188 which may be due to the length of the hydrophilic chain of PLX-188 [33,34,35,36].

In general, it is expected that upon increasing the concentration of surfactant the particle size will decrease. However, the opposite results were observed and explained by the particle aggregation due to the bridging effect of surfactant [31,32,33]. Surfactant concentration has an impact on entrapment efficiency as well. Increasing surfactant concentration decreased entrapment efficiency. The electrostatic interaction or repulsion in between surfactant and drug might cause this phenomenon. The other reason which can be assigned to this phenomenon is an increase in solubility of docetaxel in the aqueous phase in the presence of high concentration of surfactant causing it to diffuse into the aqueous environment and thus decrease the entrapment efficiency [35,36,37]. Entrapment efficiency was on a higher side for batches formulated using PLX-188 comparing to PVA [35]. PLX-188 was selected for further studies as it is known to impart stealth effect to nanoparticles [40].

In order to examine the effect of the process by which particle size reduction is achieved, batches were prepared with target of ~200 nm reducing particle size either by using the ultra-sonicator or homogenizer. Other parameters of formulation and process were kept constant i.e., amount of docetaxel (10 mg), PLGA (200 mg), surfactant concentration (0.05%), and type (PLX-188). NPs were prepared by homogenization speed of 13,500 rpm for 2 min and sonication at 60% of amplitude and time 45 s were employed to primary emulsion. Following the hardening step, particle size and PDI of NPs dispersion was measured by Malvern zeta sizer. The desired particle size (~200 nm) was obtained after sonication at 60% amplitude for 45 s (3 cycles of 15 s) as compared to homogenization. The particle size reduction method also impacted zeta potential with more favorable values obtained after sonication [20]. Hence, it was decided to adopt the sonication method to reduce particle size in the optimization studies. 

Increasing the amount of PLGA in organic solvent caused an increase in average NP size and entrapment efficiency. The effect can be understood based on viscosity of organic solvent which increases when the amount of polymer increases and results in faster solidification and thus prevents the diffusion free drug into the aqueous phase [34,35,36].

Upon increasing the drug concentration in the organic phase, the entrapment efficiency increased to a point and then declined rapidly. Increase in encapsulation efficiency can be explained as more availability of drug molecules to get entrapped in PLGA nanoparticles. After a complete consumption of PLGA available to encapsulate the drug, the drug remains un-entrapped and there is a decrease in entrapment efficiency based on the initial amount of drug taken for formulation preparation [34,35,36]. 

Vortex speed in the emulsification step and stirring speed in the hardening step did not impact the CQAs and were found insignificant in the Placket–Burmann design.

Enhanced cytoxicity was observed when docetaxel was encapsulated in the form of polymeric nanoparticles as reported in literature. This can be attributed to an increased uptake of NPs by cells [31].

In this study we have evaluated the pulmonary residence time of optimized DTX-NPs which can be useful as a non-invasive option in treatment therapy for Non-Small Cell Lung Cancer (NSCLC). Powder characterization in order to formulate a Dry Powder Inhaler which is able to deliver and deposit the formulation to deep regions of lung via inhalation device is part of our subsequent study.

## 5. Conclusions

In the presented work, we have studied the effects of formulation and process parameters on CQAs of NPs with the help of experimental design tools, such as Placket–Burmann and Box–Behnken, as part of QbD studies. Design space was created after optimization of formulation. DTX-NPs have shown increased mean residence time in lungs which is helpful for pulmonary administration of drugs. The performed work helped to understand the fundamentals of formulation and process design and the knowledge gathered can be applied in formulating a pulmonary drug delivery system based on polymeric nanoparticles to be administered by a dry powder inhaler.

## Figures and Tables

**Table 1:** Factors and their levels in Plackett–Burman experimental design.

Factors	Levels	
	Low	High
X_1_: Amount of docetaxel in organic phase (mg)	9	20
X_2_: Amount of poly (lactic-co-glycolic acid) (PLGA) in organic phase (mg)	200	300
X_3_: Concentration of surfactant in aqueous phase (%)	0.05	0.4
X_4_: Surfactant type	PLX	PVA
X_5_: Size reduction process	Homogenization (H)	Sonication (S)
X_6_: Solvent type	Ethyl acetate (EA)	Acetone (AC)
X_7_: Vortexing speed in emulsification step (rpm)	2000	3000
X_8_: Stirring speed in hardening step (rpm)	190	380

**Table 2:** Plackett–Burman design of experiment.

Formulation	X_1_ (mg)	X_2_ (mg)	X_3_ (%)	X_4_	X_5_	X_6_	X_7_ (rpm)	X_8_ (rpm)
PBD1	20	300	0.05	PLX	H	AC	2000	380
PBD2	20	200	0.4	PVA	S	EA	2000	190
PBD3	20	300	0.05	PVA	S	AC	2000	190
PBD4	9	200	0.4	PLX	S	AC	2000	380
PBD5	20	200	0.05	PLX	S	EA	3000	380
PBD6	20	200	0.4	PVA	H	AC	3000	380
PBD7	9	300	0.4	PLX	S	AC	3000	190
PBD8	20	300	0.4	PLX	H	EA	3000	190
PBD9	9	300	0.4	PVA	H	EA	2000	380
PBD10	9	300	0.05	PVA	S	EA	3000	380
PBD11	9	200	0.05	PVA	H	AC	3000	190
PBD12	9	200	0.05	PLX	H	EA	2000	190

**Table 3:** Factors with levels set in the Box–Behnken design of experiment.

Factors	Levels		
	Low	Medium	High
X_1_: Concentration of surfactant (%)	0.3	0.4	0.5
X_2_: Amount of PLGA in organic phase (mg)	100	200	300
X_3_: Number of sonication cycles	2	3	4

**Table 4:** Formulation and process variables with fixed levels.

Factor	Fixed Level
Docetaxel amount in organic phase (mg)	10
Surfactant type	PLX
Size reduction process	Sonication
Solvent type	Ethyl acetate
Vortexing speed in emulsification step (rpm)	2000
Stirring speed in hardening step (rpm)	380

**Table 5:** Box–Behnken design of experiment.

Formulation	X_1_ (%)	X_2_ (mg)	X_3_
1	0.4	300	4
2	0.5	200	2
3	0.3	300	3
4	0.4	200	3
5	0.4	300	2
6	0.4	200	3
7	0.5	100	3
8	0.3	200	2
9	0.3	200	4
10	0.4	200	3
11	0.5	200	4
12	0.4	200	3
13	0.4	100	4
14	0.4	100	2
15	0.4	200	3
16	0.5	300	3
17	0.3	100	3

**Table 6:** Results observed for Plackett–Burman Design.

Formulation	Y_1_ (nm)	Y_2_	Y_3_	Y_4_
PBD1	680	−13	75.36	0.995
PBD2	261	−10	52	0.1815
PBD3	620	−5	58	0.485
PBD4	310	−25.1	53.22	0.32
PBD5	266.9	−18.5	58.21	1.121
PBD6	590	−6	54	0.18
PBD7	380	−26.02	74.42	0.1423
PBD8	383	−22.1	58.93	0.1498
PBD9	490	−6	59	0.1282
PBD10	620	−5.5	76	0.475
PBD11	900	−3.1	71	0.155
PBD12	300	−14	77	1.273

Y_1_—average particle size; Y_2_—Zeta-potential; Y_3_—encapsulation efficiency; Y_4_—polydispersity index.

**Table 7:** Statistical Analysis—Plackett–Burman Design of experiment.

Factors	Y_1_ (nm)		Y_2_ (mV)		Y_3_ (%)		Y_4_	
	Coefficient	*p*-Value	Coefficient	*p*-Value	Coefficient	*p*-Value	Coefficient	*p*-Value
α_0_	483.41	0.0269	−12.86	0.0279	63.93	0.0200	0.47	0.0469
X_1_	−16.59	0.2563	0.43	0.1323	−4.51	0.0098	0.052	0.4253
X_2_	45.53	0.0499	−0.077	0.5508	3.02	0.0214	−0.071	0.2875
X_3_	−81.08	0.0165	−3.01	0.0190	−5.33	0.0070	−0.28	0.0082
X_4_	96.76	0.0117	6.93	0.0083	−2.26	0.0373	−0.20	0.0264
X_5_	−73.76	0.0198	−2.16	0.0265	−1.95	0.0491	e	e
X_6_	96.59	0.0117	−0.18	0.3000	e	e	−0.088	0.2064
X_7_	39.91	0.0633	−0.68	0.0842	1.50	0.0794	−0.097	0.1719
X_8_	e	e	0.51	0.1112	−1.30	0.1021	0.069	0.2987

Y_1_—average particle size; Y_2_—Zeta-potential; Y_3_—encapsulation efficiency; Y_4_—polydispersity index; α_0_—constant; X_1_—docetaxel amount in organic phase (mg); X_2_—PLGA amount in organic phase (mg); X_3_—surfactant concentration in aqueous phase (%); X_4_—surfactant type; X_5_—size reduction process; X_6_—solvent type; X_7_—vortexing speed in emulsification step (rpm); X_8_—stirrer speed in hardening step (rpm); e—Factors were not modeled by software.

**Table 8:** Results of dependent variables: Box–Behnken design.

Formulation	Y_1_ (nm)	Y_2_ (mV)	Y_3_ (%)	Y_4_
BBD1	202.2	−10.8	74.28	0.22
BBD2	296.5	−27.4	52.85	0.243
BBD3	229.8	−6.08	73.74	0.245
BBD4	233.5	−26.4	52.57	0.154
BBD5	223.1	−6.15	60.99	0.294
BBD6	259.1	−21.8	55	0.229
BBD7	420.8	34.5	37.42	0.362
BBD8	219.6	−36.7	57.5	0.135
BBD9	257.2	−27.5	82.93	0.17
BBD10	235.5	−26.8	58	0.159
BBD11	238.6	−24	61.04	0.193
BBD12	221.2	−12	49.04	0.149
BBD13	338.7	−8.34	41.43	0.222
BBD14	320.4	−18.1	42.1	0.317
BBD15	256.2	−20.8	56.1	0.226
BBD16	188.3	−14.4	56.13	0.15
BBD17	232.9	−24.1	57.92	0.103

Y_1_—average particle size; Y_2_—Zeta-potential; Y_3_—encapsulation efficiency; Y_4_—polydispersity index.

**Table 9:** Regression coefficients and *p*-values of the studied factors.

Factors	Y_1_ (nm)		Y_2_ (mV)		Y_3_ (%)		Y_4_	
	Coefficient	*p*-Value	Coefficient	*p*-Value	Coefficient	*p*-Value	Coefficient	*p*-Value
B_0_	241.10	0.0004	−21.56	0.0223	54.14	0.0028	0.18	0.0198
X_1_	25.59	0.0039	7.88	0.0373	−8.08	0.0019	0.037	0.0275
X_2_	58.67	<0.0001	−2.67	0.4133	10.78	0.0004	−0.012	0.4012
X_3_	−2.86	0.6503	2.21	0.4948	5.78	0.0106	−0.023	0.1271
X_1_X_2_	−57.35	0.0003	−16.73	0.0063	0.72	0.7690	−0.089	0.0022
X_1_X_3_	−23.88	0.0268	−1.45	0.7485	−4.31	0.1114	−0.021	0.2955
X_2_X_3_	−9.80	0.2894	−3.60	0.4347	3.49	0.1838	5.250 × 10^−3^	0.7881
$X_{1}^{2}$	4.36	0.6168	0.49	0.9105	5.52	0.0480	−0.023	0.2459
$X_{2}^{2}$	22.49	0.0307	18.55	0.0032	−3.36	0.1886	0.055	0.0202
$X_{3}^{2}$	7.51	0.3973	−7.83	0.1070	3.92	0.1332	0.025	0.2138

B_0_—constant; Y_1_—average particle size; Y_2_—Zeta-potential; Y_3_—encapsulation efficiency; Y_4_—polydispersity index; X_1_—Surfactant concentration in aqueous phase (%); X_2_—PLGA amount in organic phase (mg); X_3_—sonication cycles (number).

**Table 10:** The observed and predicted values of the optimum DTX-NP formulation based on desirability function.

Response	Observed	Predicted
Average particle size (nm)	219.6	207.8
Zeta-potential (mV)	−36.7	−36.9
Entrapment efficiency (%)	57.5	62.15
Polydispersity index	0.135	0.141

**Table 11:** Optimized lyophilization cycle.

Thermal Treatment				Primary Drying			
Step	Temperature (°C)	Time (min)	Ramp(R)/Hold (H)	Step	Temperature (°C)	Time (min)	Ramp(R)/Hold (H)
Shelf Load Temp 5 °C							
1	5	30	R	1	−20	120	R
2	−15	30	R	2	−20	1200	H
3	−15	120	H	3	0	180	R
4	−40	60	R	4	0	300	H
5	−40	300	H	5	10	60	R
				6	10	240	H
				7	30	60	R
				8	30	180	H
				Secondary drying	30	120	H

**Table 12:** Stability study results (n = 3, mean ± SD).

Parameters	Initial	Final
Particle size (nm)	210 ± 2.6	214 ± 1
PDI	0.159 ± 0.001	0.170 ± 0.001
Drug content (%)	97.1 ± 0.85	96.8 ± 0.77
Description	White powder	White powder

**Table 13:** Lung pharmacokinetic parameters.

Parameter	Free Drug	Nanoparticle Formulation
Half life (h)	1.14 ± 0.179	24.49 ± 1.19
T_max_ (h)	0.5 ± 0	0.5 ± 0
C_max_ (ng)	5011.3 ± 379.85	7050.4 ± 334.932
AUClast	7484.2 ± 1759.05	247,807.4 ± 4085.17
AUCINF_obs	10,069.6 ± 744.47	279,118.6 ± 6919.80
AUMClast	11,391.1 ± 8383.607	6,214,578 ± 206,264.9
AUMCINF_obs	21,612.4 ± 3919.255	9,578,507 ± 557,878
MRTlast	1.41 ± 0.693	25.07 ± 0.77
MRTINF_obs	2.14 ± 0.310	34.30 ± 1.37

Mean ± SD.

**Table 14:** Organ distribution of free drug and DTX nanoparticles.

	Time (h)	0.5	2	8	24	48	72
Drug	lung	53.31 ± 4.04	21.29 ± 3.20	0.589 ± 1.01	0	0	0
	liver	6.6767 ± 1.53	12 ± 4	9.97 ± 2.05	6 ± 1	1.767 ± 0.25	0.597 ± 0.04
	spleen	2.03 ± 0.45	3 ± 0.5	1.03 ± 0.25	0	0	0
	kidney	0	1.03 ± 0.30	3.23 ± 0.47	1.97 ± 0.15	1.433 ± 0.15	1 ± 0.3
NP	lung	75.00 ± 3.56	69.15 ± 2.71	61.97 ± 2.91	53.24 ± 3.64	21.81 ± 3.22	9.41 ± 0.6
	liver	4 ± 1	16 ± 3	10 ± 3	4 ± 2	1.43 ± 0.56	0.52 ± 0.02
	spleen	1 ± 0.2	2.1 ± 0.3	3 ± 0.93	0	0	0
	kidney	0	1.15	2.1 ± 0.5	1.1 ± 0.3	0.63 ± 0.57	0.47 ± 0.02

Mean ± SD.
